# Blood pressure reduction by gender and menopause status among hypertensive participants of a mobile health cardiovascular risk self-management program

**DOI:** 10.1016/j.ajpc.2025.101057

**Published:** 2025-07-16

**Authors:** Jayne Morgan, Walter Roberts, Helena Lyson, Morgan Meadows, Elizabeth Ofili, Martha Gulati, Erin D. Michos

**Affiliations:** a Hello Heart, Inc, Menlo Park, CA, USA; b Department of Cardiology, Morehouse School of Medicine, Atlanta, GA, USA; c Barbra Streisand Women’s Heart Center, Smidt Heart Institute, Cedars Sinai Medical Center, Los Angeles, USA; d Division of Cardiology, Johns Hopkins University School of Medicine, Baltimore, MD, USA

**Keywords:** Women’s health, Digital health, Hypertension, Menopause, Real world evidence

## Abstract

**Background:**

Despite being the leading cause of death for women in the United States, cardiovascular disease (CVD) in this population remains underrecognized, understudied, underdiagnosed, and undertreated. CVD risk is particularly pronounced in postmenopausal women. The current study evaluates the effectiveness of an mHealth cardiovascular risk self-management program in improving blood pressure (BP) control among women, especially during and after the onset of menopause.

**Methods:**

We used real-world data from hypertensive users of a mobile health (mHealth) application for CVD risk self-management to evaluate sex differences in treatment responses. We further compared BP reductions by menopause status for a subset of users whose menopause status was known.

**Results:**

A total of 48,121 participants were included in the analysis (mean age: 51.8 ± 11.0 years, 55.3 % women, mean baseline systolic BP: 135.5 ± 17.3 mmHg, mean baseline diastolic BP: 85.3 ± 11.6 mmHg). The primary analysis found that women users showed a larger reduction in BP compared to men (*p* < 0.05). Although perimenopausal and postmenopausal women started with higher baseline BP than premenopausal women (*p* < 0.001), all groups showed comparable reductions in systolic BP.

**Conclusion:**

There were clinically meaningful reductions in BP among participants of an mHealth app, with women showing greater BP reductions than men. Findings suggest that targeted, sex-specific health information and digital coaching can be effective in reducing cardiovascular risk among women during and after menopause.

## 1 Introduction

Cardiovascular disease (CVD) affects >60 million women in the U.S. and is the leading cause of death for women globally [1] Despite this, CVD among women is notably underrecognized, understudied, underdiagnosed, and undertreated [2] Women face persistent disparities in CVD compared to men that stem from a myriad of interrelated factors, including less aggressive management of CVD and CVD risk factors and underrepresentation in clinical trials [3] These disparities are even more pronounced among racial and ethnic minority groups [4] There also are sex-specific risk factors, in particular a nearly threefold increase in CVD risk during menopause[5] that is often attributed to mid-life withdrawal of endogenous sex steroids associated with the menopausal transition [6] The prevalence of metabolic syndrome also increases with menopause, and the prevalence of hypertension increases with age [7] Taken together, these disparities underscore an urgent need for tailored interventions that address the unique cardiovascular risk and barriers to adequate diagnosis and treatment faced by women during this time period.

Widespread smartphone ownership among U.S. adults[8] has created promising opportunities for digital health interventions, particularly smartphone-based mobile health (mHealth) programs, to enhance cardiovascular risk self-management and improve clinical outcomes for broad segments of the population by reducing geographical, structural, and economic barriers to care. By providing on-demand, easily accessible healthcare services and information, mHealth programs can be particularly effective at reaching individuals who may be less likely to engage with traditional healthcare settings due to adverse social determinants of health [9] Importantly, mHealth cardiovascular risk self-management programs have shown promise in helping individuals manage high blood pressure (BP)–the most common risk factor for CVD[10] –and other cardiovascular risk factors such as cholesterol, diabetes and weight[11], with subsequent reduction in the risk of CVD events, all-cause mortality, and hospitalizations [12] Moreover, mHealth programs that provide personalized, evidence-based education, promote healthy lifestyle choices through digital coaching, and facilitate communication with healthcare professionals can be tailored and leveraged to address cardiovascular risk specific to certain demographic groups, such as pregnant/postpartum or menopausal women [9] For example, previous research found an mHealth, SMS-based tailored intervention to have a positive clinical impact on weight, cholesterol, and BP among a population of postmenopausal women with abdominal obesity[13], underscoring the clinical effectiveness of targeted mHealth interventions to address cardiovascular risk factors in high-risk populations.

Further research is needed to more thoroughly assess the clinical effectiveness of mHealth programs across diverse settings and populations, particularly among women during periods of heightened cardiovascular risk in their lives, such as during and after menopause. Towards that end, this study aims to evaluate the clinical effectiveness of an mHealth cardiovascular risk self-management program in improving cardiovascular risk through BP control among women, especially during and after menopause.

## 2 Methods

This study is a retrospective cohort observational analysis of participants enrolled in the Hello Heart (Hello Heart, Inc, Menlo Park, CA) mHealth cardiovascular risk self-management program from July 2015 to September 2023.

### 2.1 Intervention description

The mHealth program consists of a mobile application (app) and a Bluetooth-enabled BP monitor (Zewa UAM-906HH, Zewa UAM‐905HH, or A&D UA‐651BLE) that provides heart health-related metrics tracking (e.g. BP, heart rate, cholesterol, weight), medication tracking and adherence reminders, artificial intelligence-driven and evidence-based personalized lifestyle digital coaching, personalized health reports, and clinically-based, sex-specific user flows. This includes tailored educational content and digital coaching for menopausal women to increase awareness and understanding of how menopause impacts cardiovascular risk and ways to manage this risk. Additionally, the recommendations align with established guidelines[14], and are adapted to individual user preferences. While there may be differences in the user experience due to personalized adaptations, all users receive content that is guidelines-based but tailored to their personal characteristics [15] This approach is designed to maximize the likelihood of positive health behavior changes across the user population. A more comprehensive description of the program has been previously published [11]

### 2.2 Participants

Study participants included individuals ≥18 years of age with hypertension, defined as a prior diagnosis of hypertension and/or relevant medical/pharmacy insurance claim related to hypertension, who enrolled voluntarily through their (or their spouse’s/domestic partner’s) employer-based health plan. Each employer provided initial access to the program for their employees at different times, and enrollment occurred on an ongoing basis. Participants enrolled in response to mailed postcards, emails, onsite enrollment communications, or through the employer’s benefits package online portal communications. All individuals who enrolled in the program, for any length of enrollment time, from an eligible employer, were eligible for inclusion in the study. Participants were included in the analysis if they provided two or more BP measurements in the app, including one during the baseline period as defined below. Potential participants were excluded if they did not meet the inclusion criteria. Participants were employed across a range of industries (e.g., manufacturing, public sector, retail). The geographical distribution of users is visualized in Supplemental Figure 1.

**Fig. 1:**
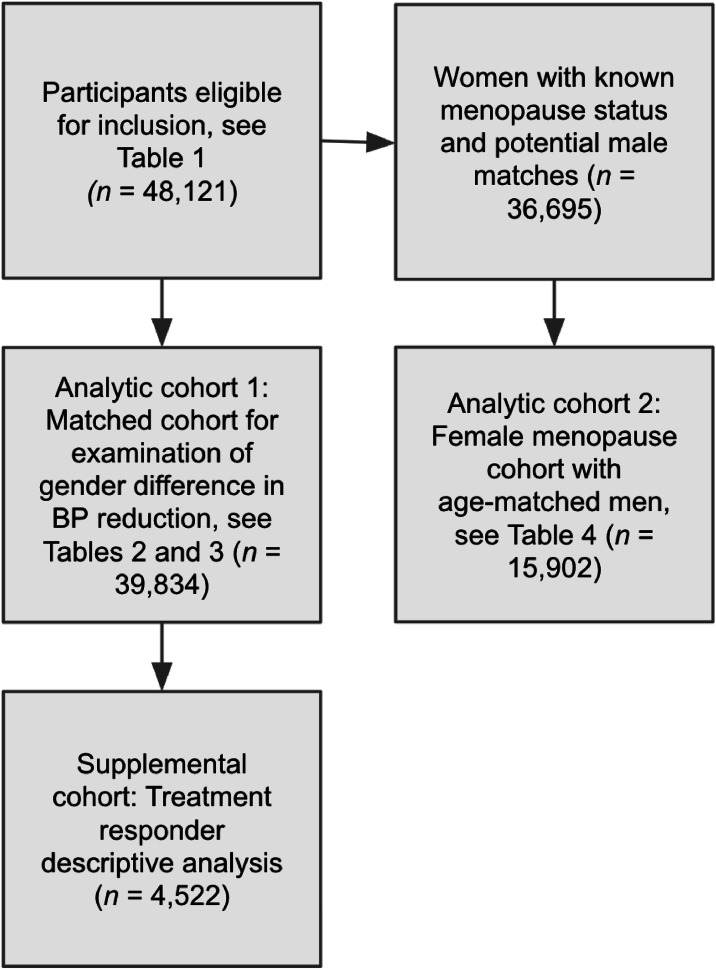
Cohort definitions and participant flow.

Three analytic cohorts were derived as illustrated in Fig. 1. First, to examine sex differences, we used a 1:1 coarsened exact matching procedure that required complete demographic data. Second, we formed a menopause cohort limited to users ≥45 years of age who self-identified as pre-, peri‑, or postmenopausal, along with an age-matched men comparison group, in order to assess potential differences in BP across sex and menopause status. Third, we completed an analysis of treatment responders who achieved a systolic BP reduction.

All participants consented to terms permitting research use of de-identified, encrypted data. This study was reviewed by the Western Copernicus Group Institutional Review Board (IRB tracking ID 20,226,635) and determined to be exempt under 45 CFR 46.104(d)(4). In addition, a waiver of HIPAA authorization for the use and disclosure of aggregated, de-identified data was obtained. No compensation was provided to participants.

### 2.3 Measures

Participants provided self‐reported demographic data (age, sex, geographic location, and relationship as employee or spouse/domestic partner) and selected clinical data (diabetes diagnosis, antihypertensive medication usage) at the time of downloading the application. Diabetes diagnosis and antihypertensive medication prescription were self‐reported and, as such, may underestimate the true prevalence of these conditions in the sample.

The primary outcome measure in the study was monthly BP as self-measured by participants using the Bluetooth-enabled BP monitors described above. Importantly, the app provides both video and text-based coaching on best practices for BP measurement (e.g., appropriate seating position), consistent with AHA and other guidelines. Participants can set notifications prompting BP measurement, with a default of daily notifications. Monthly BP was calculated as the median systolic and diastolic BP across all measurements that occurred during the respective month. Baseline BP was calculated as median systolic and diastolic BP during the first week of BP measurement after program enlistment. Occurrence of hypertensive crises was considered as a secondary outcome. A hypertensive crisis was defined as 2 consecutive readings of > 180 mmHg systolic and/or > 120 mmHg diastolic.

### 2.4 Statistical approach

Analyses were performed in R Version 4.4.1. We first examined BP reduction over the initial one year of program participation. Changes in BP over 12 months were analyzed using linear mixed models to characterize reductions over time and to test the significance of sex interactions. To better isolate any sex differences in clinical outcome, we then utilized 1:1 coarsened exact matching (CEM) to ensure similar baseline characteristics between men and women [16] Participants were matched on self-reported antihypertensive medication status, age group, and baseline systolic BP. Clinical outcomes evaluated with this approach included systolic BP, diastolic BP, and hypertensive crisis episodes (defined as BP>180/120 mmHg).

The models included fixed effects for measurement month, age group, self-reported antihypertensive medication status, and sex, with random intercepts specified for each participant. The interaction between sex and time was included as the critical test of sex differences in BP reduction. All available data were included in the models, as linear mixed-effects models inherently handle missing data through maximum likelihood estimation. For binary outcomes (i.e., hypertensive crisis events with BP > 180/120 mmHg), we used logistic models with a logit link function. All continuous variables were standardized in the models.

As a sensitivity analysis, we imputed missing data using a last observation carried forward approach. The critical parameter estimates involving sex were not substantively different in these analyses, so we present the models estimated using all available data without imputation.

For the analysis of the menopause cohort, we first tested for baseline differences in systolic BP according to participants' menopause status. To enhance this comparison and highlight sex differences, we identified a 1:1 age-matched group of men to compare against the menopause cohorts. Men were assigned to a menopause-matched group based on the menopause status of their age-indexed matched female counterpart. This approach allowed us to compare the magnitude of the sex differences in BP at different stages of menopause, effectively quantifying the changes in relative risk at each stage of menopause. These data were analyzed using a general linear model (GLM) with baseline BP as a dependent variable and menopause status, self-reported antihypertensive medication status, and self-reported diabetes diagnosis as independent covariates.

We then tested for differences in BP reductions related to menopause status using a linear mixed model with BP as dependent variable and menopause status, self-reported antihypertensive medication status, self-reported diabetes diagnosis, and measurement time as independent variables. The interaction between menopause status and time was included as the critical test of this hypothesis. As a sensitivity analysis, we included age in these models, and its inclusion did not change the significance of the critical terms.

We also conducted a supplemental descriptive analysis to evaluate sex differences in the magnitude of systolic BP reduction among treatment responders (defined as participants whose median systolic BP at the point of reading was lower than their baseline) with baseline systolic BP ≥140 mmHg. Participants were only included in this cohort if they took one or more BP readings between weeks 50 and 54. Participants engaged beyond this follow-up period were not included in the treatment responder analysis unless they also collected BP measurements during the pre-specified follow up time period. For this analysis, we elected to classify participants’ baseline BP using only systolic BP in keeping with evidence that systolic BP is a stronger predictor of cardiovascular risk than diastolic BP, particularly in women [17] Additionally, systolic BP is a key input for different atherosclerotic cardiovascular disease risk calculators and is more strongly associated with cardiovascular complications and mortality [18] Results of this analysis are presented in supplemental Table 2.

## 3 Results

### 3.1 Descriptive characteristics

A total of 48,121 participants were eligible for inclusion in the analysis (mean age: 52.6 years (SD = 10.65), 55.1 % women, mean baseline systolic BP: 135.5 mmHg (SD = 16.9), mean baseline diastolic BP: 85.2 mmHg (SD = 11.4)). Descriptive statistics are reported separately by sex in Table 1. Descriptions of the full and matched sample are provided in Table 2.

**Table 1:** Clinical and demographic characteristics of eligible users at baseline.

	Women (*n* = 26,537)	Men (*n* = 21,584)
	M (SD), %[n]	M (SD), %[n]
Baseline Systolic BP, mmHg	134.1 (17.3)	137.2 (16.1)
Baseline Diastolic BP, mmHg	84.9 (11.5)	85.6 (11.1)
Age (years)	52.6 (10.3)	52.6 (11.0)
Diabetes ( %)	8.8 [2325]	8.3 [1801]
≥1 hypertensive crisis reading >180/120 mmHg ( %)	5.5 [1469]	5.1 [1096]
Baseline Categorical Systolic BP		
120–129 mmHg Systolic	24.5 [6507]	20.1 [4341]
130–139 mmHg Systolic	32.2 [8551]	31.7 [6839]
140+ mmHg Systolic	43.3 [11,479]	48.2 [10,404]

**Table 2:** Description of the sample before and after coarsened exact matching procedure applied.

	Eligible Users (*n* = 48,121)		Matched sample (*n* = 39,834)	
	Men	Women	Men	Women
N	21,584	26,537	19,917	19,917
Age (years)	52.6	52.6	52.7	52.7
Baseline Systolic BP, mmHg	137.2	134.1	135.3	135.3
Baseline Diastolic BP, mmHg	85.6	84.9	85.2	84.2
Antihypertensive medication % (self-report)	30.0	38.7	31.7	31.7
Diabetes % (self-report)	8.3	8.8	8.2	8.8

### 3.2 Sex differences in BP reductions

Model parameter estimates are presented in Table 3, and BP readings by month are reported in Supplemental Table 1. Estimates support reductions in systolic BP pressure over time, *B* = −0.04, *SE* = 0.002, *t* = −21.4, *p* < 0.001. Importantly, there was a significant interaction between time and genderx, *B* = 0.01, *SE* = 0.003, *t* = 2.19, *p* = 0.029, indicating that women had a larger reduction in systolic BP over time. Simple slope analysis confirmed a larger reduction over time in women, *B* = −0.18, compared to men, *B* = −0.15. Findings were similar for diastolic BP, including the significantly larger reduction over time for women.

**Table 3:** Linear mixed models evaluating sex differences in clinical outcomes over time.

		*B*	*SEB*	*t (z)*	***p***
Systolic BP (mmHg)					
	Month	−0.043	0.002	−21.43	<0.001
	Gender (man)	0.041	0.008	4.87	<0.001
	Gender X Month	0.006	0.003	2.19	0.029
	Age (ref = < 35 years)				
	35 to 45	0.074	0.020	3.85	<0.001
	46 to 55	0.172	0.018	9.30	<0.001
	55 to 65	0.192	0.018	10.56	<0.001
	66+	0.223	0.022	10.12	<0.001
	Antihypertensive medication use	0.205	0.009	22.93	<0.001
	Diabetes	0.197	0.015	13.15	<0.001
Diastolic BP (mmHg)					
	Month	−0.051	0.002	−25.70	< 0.001
	Gender (man)	−0.069	0.008	−8.27	< 0.001
	GenderX Month	0.008	0.003	2.93	0.003
	Age (ref = < 35 years)				
	35 to 45	0.216	0.019	11.32	< 0.001
	46 to 55	0.257	0.018	13.99	< 0.001
	55 to 65	0.034	0.022	1.88	0.060
	66+	−0.25	0.009	−18.23	< 0.001
	Antihypertensive medication use	0.162	0.009	18.23	< 0.001
	Diabetes	0.003	0.015	0.23	0.820
Hypertensive Crisis (count)					
	Month	−0.0.078	0.005	(−15.24)	< 0.001
	Gender (man)	−0.317	0.042	(−7.53)	< 0.001
	Gender X Month	−0.003	0.008	(−0.33)	0.742
	Age (ref = < 35 years)				
	35 to 45	0.064	0.088	(0.73)	0.467
	46 to 55	0.041	0.084	(0.49)	0.623
	55 to 65	0.080	0.083	(0.97)	0.332
	66+	0.205	0.093	(2.21)	0.027
	Antihypertensive medication use	0.157	0.034	(4.70)	< 0.001
	Diabetes	0.310	0.053	(5.85)	< 0.001

Note. All continuous variables were standardized.

There was a significant effect of time on the likelihood of hypertensive crisis, with decreasing risk of hypertensive crisis over time, *B* = −0.21, *SE* = 0.005, *z* = −41.55, *p* < 0.001. The interaction between time and sex was not significant, indicating no differential effect of sex on likelihood of hypertensive crisis, *B* = 0.003, *SE* = 0.008, *z* = −0.33, *p* = 0.742.

### 3.3 Examination of baseline risk and treatment effects across menopause stages

**Fig. 2:**
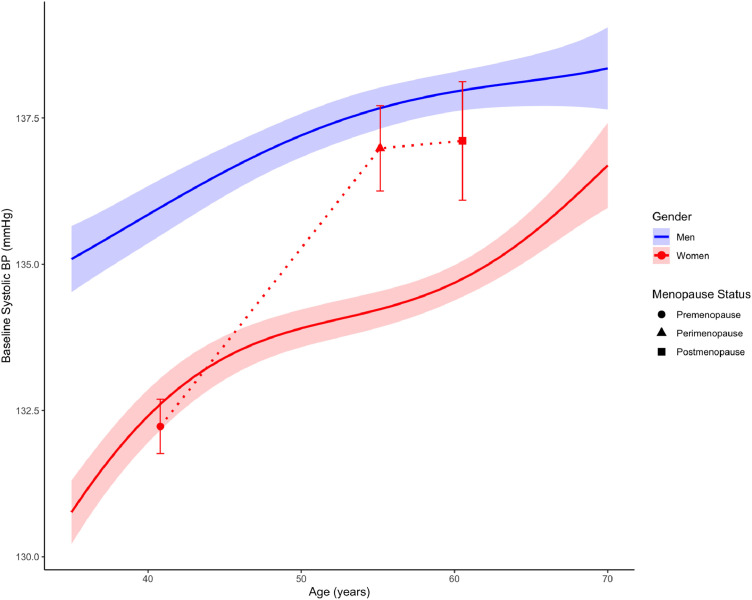
Predicted baseline systolic blood pressure (SBP) by age and sex, highlighting menopausal status among women. The solid lines (blue for men, red for women)represent modeled SBP estimates with shaded 95 % confidence bands. For women, distinct symbols—circle (premenopause), triangle (perimenopause), and square (postmenopause)—show observed mean SBP with 95 % confidence interval error bars. Perimenopausal and postmenopausal women exhibit higher baseline SBP than premenopausal women when compared at similar ages.

Baseline characteristics of the menopause cohort and the age-matched index sample of men is shown in Table 4. The cohort included 5196 women identified as being in premenopause, 1886 in perimenopause, and 1062 in postmenopause. The GLM examining baseline systolic BP found a significant interaction between sex and perimenopause status (*B* = −0.19, *SEB* = 0.038, *t* = −4.96, *p* < 0.001) and sex and postmenopausal status *(B* = −0.15, *SEB* = 0.049, *t* = −3.03, *p* = 0.002), indicating higher baseline systolic BP in perimenopausal and postmenopausal women relative to their premenopausal counterparts when compared to age-matched men. Hypertensive crises were more likely in the peri‑ and postmenopausal women as compared to premenopausal women (all *p* < 0.05), however there was no significant interaction with sex when compared to age-matched men (all *p* > 0.05). Fig. 2 visualizes average baseline systolic BP for each menopause status group. This figure also illustrates the linear association between age and baseline BP separately in men and women. As seen in this figure, self-report of peri‑ and post-menopause status correspond to higher baseline systolic BP than would be predicted by age alone.

**Table 4:** Baseline characteristics of the menopause cohorts and age-matched men.

	Premenopause		Perimenopause		Postmenopause	
	Women	Age-matched Men	Women	Age-matched Men	Women	Age-matched Men
	M ( %)	M ( %)	M ( %)	M ( %)	M ( %)	M ( %)
Age (years)	40.80	41.42	55.15	55.16	60.52	60.52
≥ 1 hypertensive crisis	(5.09)	(3.6)	(5.43)	(5.34)	(5.54)	(5.03)
Antihypertensive medication % (self-report)	(30.49)	(23.42)	(56.26)	(31.66)	(56.41)	(33.54)
Diabetes % (self-report)	(6.63)	(5.78)	(11.35)	(12.02)	(9.95)	(12.10)

The linear mixed model examining changes in systolic BP over time found no significant interaction between time and perimenopausal status (*B* = 0.002, SEB = 0.002, *t* = 0.80, *p* = 0.421) or time and postmenopause status (*B* = −0.003, *SEB* = 0.002, *t* = −1.56, *p* = 0.119), in both cases as compared to premenopause status. Similarly, there was no significant interaction between time and menopause status for diastolic BP or hypertensive crisis outcomes (all *p* > 0.05). These findings suggest that the intervention was similarly effective at reducing BP across menopause groups. The supplemental examination of gender differences in BP reduction among treatment responders is shown in Supplemental Table 2.

## 4 Discussion

This study demonstrates clinically meaningful reductions in BP among a large population of participants with hypertension using a mHealth cardiovascular risk self-management program. These results align with previous research showing substantial BP reductions in other large cohorts of participants using this same mHealth program [11,19] Reductions of this magnitude are clinically meaningful, as prior studies have reported that a 5‐mm Hg reduction of systolic BP reduced the risk of major cardiovascular events by about 10 %, while a 10‐mm Hg reduction of systolic BP reduced the risk of major cardiovascular events by 20 % [[20], [21], [22], [23], [24]]

The current analysis adds to prior studies on cardiovascular mHealth interventions by demonstrating that women experienced greater BP reductions than their male counterparts. This is notable because prior research suggests that women have poorer BP control than men over time, and that women may be less adherent to antihypertensive and antilipidemic medications due to side effects [25] These results also suggest that sex may play an important role in clinical responsiveness to lifestyle-based, behavioral mHealth interventions, and are consistent with prior research that has shown that women are more likely to seek technology-based health information and use mHealth apps as compared to men [[26], [27], [28]] While alternative research methodologies (i.e., randomized clinical trials) will be important to provide more precise estimates of sex differences in expected treatment effects, we evaluated the sex difference in the magnitude of reduction in a clinically relevant subgroup of users (i.e., users who started with baseline systolic BP ≥140 mmHg, remained engaged for the full year, and responded to the treatment) [29] This analysis found that women in this cohort showed an additional 1.2 points reduction relative to comparable men. Future research should explore the mechanisms contributing to these greater clinical benefits for women compared to men.

Additionally, evaluation of BP changes across menopause stages revealed that women in perimenopause and postmenopause had higher baseline systolic BP relative to their premenopausal counterparts, as well as higher likelihood of prior hypertensive crisis occurrence. This finding echoes previous research demonstrating that women face increased cardiovascular risk during menopause[5], and reinforces the need for targeted, sex-specific interventions that address the unique health challenges faced during this critical life stage [7] Despite this elevated baseline risk, our results indicate that all menopause groups achieved similar reductions in systolic BP after 1 year of program participation. The mHealth intervention evaluated in this study includes tailored educational content and digital coaching for menopausal women s to increase awareness and understanding of how menopause impacts cardiovascular risk and ways to manage this risk, and the women in the menopause cohort did access this functionality. These results provide promising evidence that tailored mHealth content and digital coaching can effectively support cardiovascular risk management for higher-risk, peri‑ and postmenopausal women, and that these targeted interventions can ultimately help ameliorate significant disparities in cardiovascular health and outcomes. Additional research is needed to further explore the specific mechanisms within the mHealth program contributing to these improvements and to understand how stage of menopause may influence intervention effectiveness.

Our study has several limitations. As this was an observational study, one cannot make a causal inference based on the results. The generalizability of our findings are limited given that the study population was middle-aged individuals with employer-sponsored health insurance and participants elected to enroll in the program. Although no incentives were provided for enrollment in the program, it is also possible that participants were more interested or motivated to improve their health and could thus contribute to a selection effect in the study population. We relied on self-reported data for key clinical and demographic variables, including antihypertensive medication use, diabetes diagnosis, and menopause status. This approach is subject to recall bias and social desirability bias, which may lead to misclassification. For instance, the self-identification of menopausal status is subjective and may not perfectly align with a formal clinical diagnosis, potentially affecting the precision of our findings related to this subgroup. Furthermore, while the primary outcome of BP was captured via a connected device, the measurements were self-administered. Although the app provides coaching on proper measurement technique, we cannot guarantee user adherence to these guidelines for every reading, which could introduce variability into the BP data.

Menopause status was only collected for a subset of participants, and more granular data such as age at menopause, years since menopause, or use of menopausal hormone therapy, was not available. We also did not collect race/ethnicity or socioeconomic data from participants, which prevented us from evaluating outcomes by these key sociodemographic factors. This limitation hinders our ability to address important issues of health equity and the effectiveness of mHealth interventions, particularly concerning the disparate CVD health outcomes faced by women from racial and ethnic minority groups and those from low-income populations. Future research should assess the impact of social determinants of health on clinical outcomes to better understand how mHealth programs can be tailored and implemented in culturally resonant, accessible, and clinically effective ways across diverse populations. Nevertheless, key strengths of our study include a large population of participants, situated in geographically diverse locations across a variety of blue- and white-collar industries, with long-term, real-world follow-up data. This broad representation in our study population enhances the generalizability of our findings, and our results underscore the significant clinical impact of mHealth programs in cardiovascular risk self-management.

## 5 Conclusion

By demonstrating clinically meaningful reductions in BP among a large population of participants of an mHealth cardiovascular risk self-management program, this study contributes valuable empirical data supporting the effectiveness of mHealth interventions for hypertension management. Our findings also indicate that women showed greater BP reductions than men, suggesting that sex may influence responsiveness to tailored, lifestyle-based interventions. Additionally, the higher baseline BP observed in peri‑and post-menopausal women compared to premenopausal women reinforces the previously established heightened cardiovascular risk women face during specific periods of their lives, and underscores the need for targeted, sex-tailored strategies to address these health challenges. Our results highlight the potential effectiveness of targeted, sex-specific health information and digital coaching to address cardiovascular risk in womens during this crucial life stage. Overall, this study supports the potential of mHealth technology to empower individuals to effectively self-manage cardiovascular risk factors, especially during critical periods of heightened cardiovascular risk for women.

## Author declarations

We wish to draw the attention of the Editor to the following facts which may be considered as potential conflicts of interest and to significant financial contributions to this work. Jayne Morgan reports financial support was provided by Hello Heart, Inc. Morgan Meadows reports financial support was provided by Hello Heart, Inc. Walter Roberts reports financial support was provided by Hello Heart, Inc. Helena Lyson reports financial support was provided by Hello Heart, Inc. Martha Gulati reports financial support was provided by Hello Heart, Inc. Jayne Morgan has served as an adviser for Midi Health. Erin Michos has served as a consultant for Amgen, Arrowhead, AstraZeneca, Bayer, Boehringer Ingelheim, Edwards Life Science, Esperion, Ionis, Eli Lilly, Medtronic, Merck, New Amsterdam, Novartis, Novo Nordisk, and Zoll. Elizabeth Ofili has served as an adviser for Mineralys, founded Accuhealth Technologies Inc, and serves as a global Principal Investigator for the African American Heart Study (Amgen). Martha Gulati has served as a consultant for Novartis and Eserion and has received speaking fees from Siemans.

We confirm that the manuscript has been read and approved by all named authors and that there are no other persons who satisfied the criteria for authorship but are not listed. We further confirm that the order of authors listed in the manuscript has been approved by all of us. We confirm that we have given due consideration to the protection of intellectual property associated with this work and that there are no impediments to publication, including the timing of publication, with respect to intellectual property. In so doing we confirm that we have followed the regulations of our institutions concerning intellectual property. We further confirm that any aspect of the work covered in this manuscript that has involved either experimental animals or human patients has been conducted with the ethical approval of all relevant bodies and that such approvals are acknowledged within the manuscript.

We understand that the Corresponding Author is the sole contact for the Editorial process (including Editorial Manager and direct communications with the office). She is responsible for communicating with the other authors about progress, submissions of revisions and final approval of proofs. We confirm that we have provided a current, correct email address which is accessible by the Corresponding Author.

**Figure:**
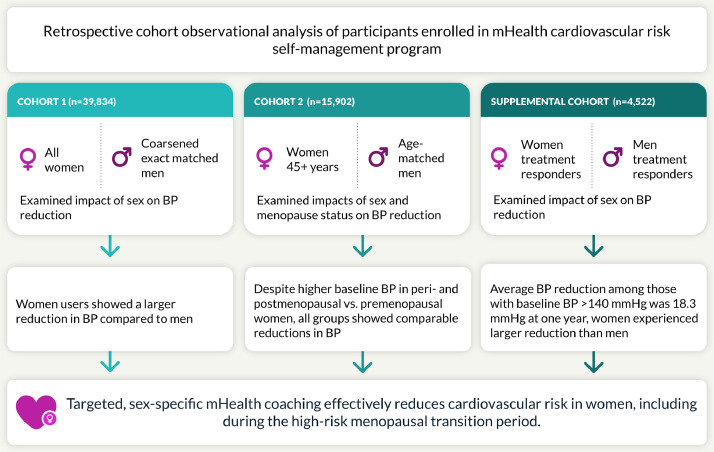
Central illustration. graphical representation of study design.

## Funding

Funding for this study was provided by Hello Heart.

## CRediT authorship contribution statement

**Jayne Morgan:** Writing – review & editing, Supervision, Conceptualization. **Walter Roberts:** Writing – review & editing, Formal analysis, Data curation. **Helena Lyson:** Writing – original draft, Project administration. **Morgan Meadows:** Formal analysis, Data curation. **Elizabeth Ofili:** Writing – review & editing. **Martha Gulati:** Writing – review & editing. **Erin D. Michos:** Writing – review & editing, Supervision.

## Declaration of competing interest

Jayne Morgan reports financial support was provided by Hello Heart, Inc. Morgan Meadows reports financial support was provided by Hello Heart, Inc. Helena Lyson reports financial support was provided by Hello Heart, Inc. Walter Roberts reports financial support was provided by Hello Heart, Inc. Martha Gulati reports financial support was provided by Hello Heart, Inc. Jayne Morgan has served as an adviser for Midi Health. Erin Michos has served as a consultant for Amgen, Arrowhead, AstraZeneca, Bayer, Boehringer Ingelheim, Edwards Life Science, Esperion, Ionis, Eli Lilly, Medtronic, Merck, New Amsterdam, Novartis, Novo Nordisk, and Zoll. Elizabeth Ofili has served as an adviser for Mineralys, founded Accuhealth Technologies Inc, and serves as a global Principal Investigator for the African American Heart Study (Amgen). Martha Gulati has served as a consultant for Novartis and Eserion and has received speaking fees from Siemans.

## Appendix Supplementary materials

Image, application 1

Image, application 2

Image, application 3
