# Supplementary figures and images for: Blood pressure reduction by gender and menopause status among hypertensive participants of a mobile health cardiovascular risk self-management program
Source: Am J Prev Cardiol. 2025 Jul 16;23:101057. doi: 10.1016/j.ajpc.2025.101057 (PMC12309255; doi:10.1016/j.ajpc.2025.101057)

Figure 1. Geographical distribution of participants across the United States


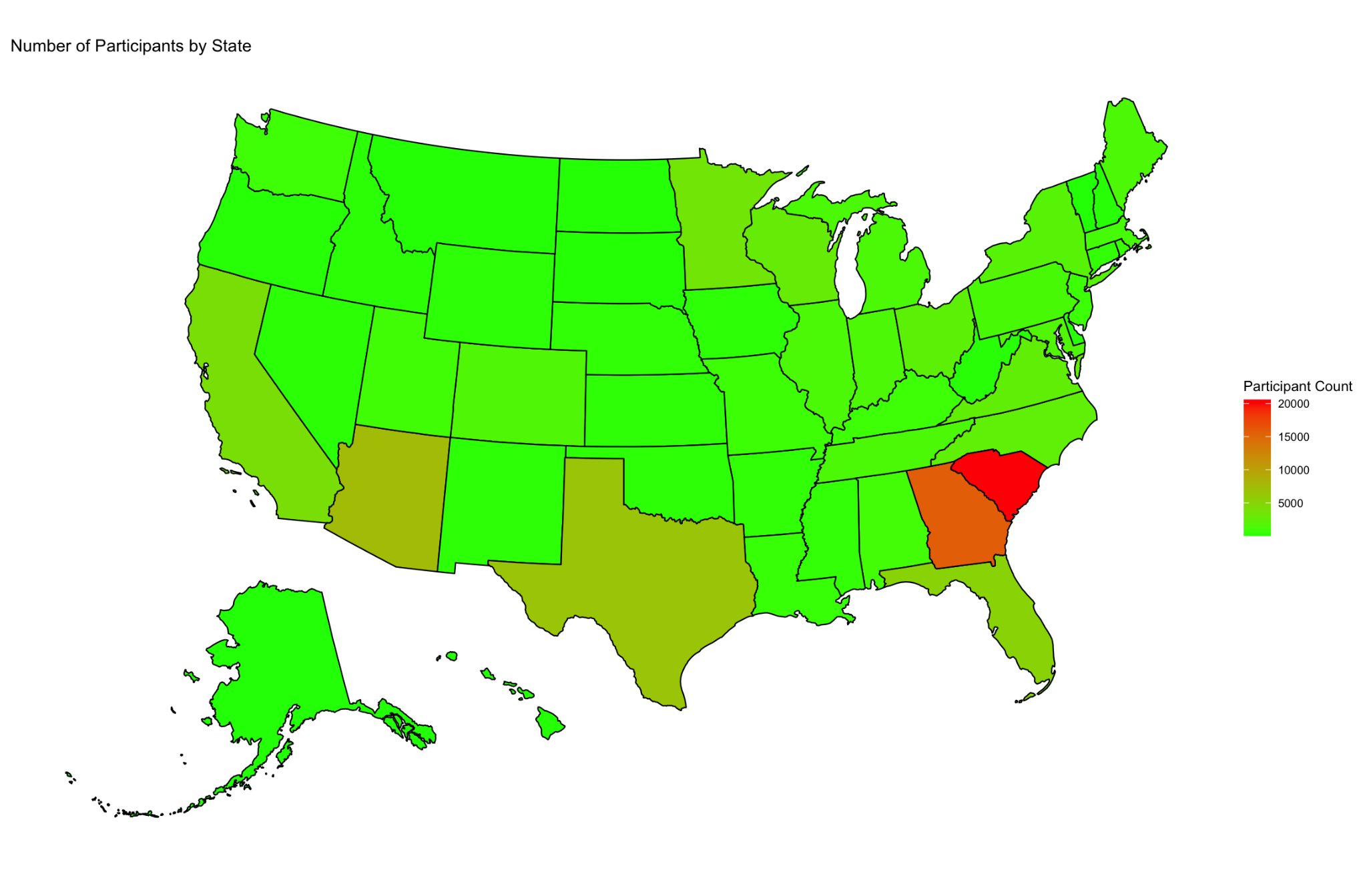

Supplement: Supplementary file 1 [file mmc1.docx]
