# Supplementary material for: Blood pressure reduction by gender and menopause status among hypertensive participants of a mobile health cardiovascular risk self-management program
Source: Am J Prev Cardiol. 2025 Jul 16;23:101057. doi: 10.1016/j.ajpc.2025.101057 (PMC12309255; doi:10.1016/j.ajpc.2025.101057)
Supplement: Supplementary file 2 [file mmc2.docx]

| Supplemental Table 1. Mean number of blood pressure readings each month following enrollment | | |
| --- | --- | --- |
|  | Mean (SD) Monthly Measurements | |
| Month | Men | Women |
| 1 | 20.1 (26.0) | 16.5 (21.7) |
| 2 | 15.0 (22.9) | 12.7 (21.3) |
| 3 | 13.6 (22.2) | 11.9 (19.7) |
| 4 | 13.0 (21.5) | 11.3 (18.4) |
| 5 | 12.6 (22.4) | 11.0 (17.6) |
| 6 | 12.6 (24.4) | 10.8 (17.4) |
| 7 | 12.2 (21.8) | 10.4 (16.7) |
| 8 | 11.9 (21.0) | 10.4 (17.1) |
| 9 | 11.7 (21.6) | 10.5 (17.9) |
| 10 | 11.6 (20.5) | 10.5 (19.5) |
| 11 | 11.4 (19.3) | 10.0 (16.4) |
| 12 | 11.7 (19.2) | 10.4 (16.6) |
