# Supplementary material for: Blood pressure reduction by gender and menopause status among hypertensive participants of a mobile health cardiovascular risk self-management program
Source: Am J Prev Cardiol. 2025 Jul 16;23:101057. doi: 10.1016/j.ajpc.2025.101057 (PMC12309255; doi:10.1016/j.ajpc.2025.101057)
Supplement: Supplementary file 3 [file mmc3.docx]

| Supplemental Table 2. Mean Observed Reductions in Systolic Blood Pressure among Treatment Responders with > 140 mmHg Systolic at Baseline | | |
| --- | --- | --- |
|  | Baseline Systolic  M (SD) | Month 12 Reduction Systolic M (SD) |
| Women | 152.9 (11.3) | 19.1 (13.1) |
| Men | 152.4 (10.9) | 17.6 (12.5) |
| Combined | 150.7 (11.1) | 18.3 (12.8) |
